# Restructuring Primary Health Care Markets in New Zealand: from Welfare Benefits to Insurance Markets

**DOI:** 10.1186/1743-8462-2-20

**Published:** 2005-09-06

**Authors:** Bronwyn Howell

**Affiliations:** 1 New Zealand Institute for the Study of Competition and Regulation; and Victoria Management School, Victoria University of Wellington, Rutherford House, 23 Lambton Quay, Wellington, New Zealand

## Abstract

**Background:**

New Zealand's Primary Health Care Strategy (NZPHCS) was introduced in 2002. Its features are substantial increases in government funding delivered as capitation payments, and newly-created service-purchasing agencies. The objectives are to reduce health disparities and to improve health outcomes.

**Analysis:**

The NZPHCS changes New Zealand's publicly-funded primary health care payments from targeted welfare benefits to universal, risk-rated insurance premium subsidies. Patient contributions change from fee-for-service top-ups to insurance premium top-ups, and are collected by service providers who, depending upon their contracts with purchasers, may also be either insurance agents or risk-bearing insurance companies. The change invokes the tensions associated with allocating risk-bearing amongst providers, patients and insurance companies that accompany all insurance-based funding instruments. These include increases in existing incentives for over-consumption and new incentives for insurers to limit their exposure to variations in patient health states by engaging in active patient pool selection.

The New Zealand scheme is complex, but closely resembles United States insurance-based, risk-rated managed care schemes. The key difference is that unlike classic managed care models, where provider remuneration is determined by the insurer, the historic right for general practitioners to autonomously set patient charges alters the fiscal incentives normally available to managed care organisations. Consequently, the insurance role is being devolved to individual service providers with very small patient pools, who must recoup the premium top-ups from insured individuals. Premium top-ups are being collected only from those individuals consuming care, in proportion to the number of times care is sought. Co-payments thus constitute perfectly risk-rated premium levies set by inefficiently small insurers, raising questions about the efficiency and equity of a 'universal' insurance system pooling total population demands and costs. The efficacy of using financial incentives to constrain costs and encourage innovation when providers retain the right to arbitrarily recoup costs directly from patients, is also questioned.

**Results:**

Initial evidence suggests that total costs are higher than initially expected, and prices to some patients have risen substantially under the NZPHCS. Limited competition and NZPHCS governance requirements mean current institutional arrangements are unlikely to facilitate efficiency improvements. System design changes therefore appear indicated.

## Background

New Zealand's state-funded primary health care system has undergone fundamental structural and financial change following the implementation of the Primary Health Care Strategy (NZPHCS), beginning in 2002 [1]. The principal stated objectives of the NZPHCS are to reduce health disparities, to improve health outcomes and to increase the share of government funding in primary care. In pursuit of these objectives, the NZPHCS utilises two financial instruments and a structural instrument.

The financial instruments are an increase in the quantum of taxpayer funds applied to primary health care (a 43% increase in the first three years [2], with $1.7 billion additional funds being applied over 6 years [3]), and universal capitation funding for all enrolled citizens, irrespective of individual consumption of primary health care services. Whilst all citizens are eligible to receive capitation funding, the rate paid varies depending upon patient characteristics such as age, gender, ethnicity and financial deprivation, and the characteristics of the entity to which the capitation subsidies are paid (see Table 1).

**Table 1:** PHO Types and Annual Capitation Subsidies, 2004–5 [67]

**Capitaiton Subsidies**										
		**GMS/Nurse**					**Services to Improve Access**			
**PHO Type**		**Interim**			**Access**		**All**			

			**HUHC**		**HUHC**		**Maori/Pacific**		**Non Maori/Pacific**	
**Age Group**	**Gender**	**CSC**	**N**	**Y**	**N**	**Y**	**1 thru 4**	**5**	**1 thru 4**	**5**

00–04	F	Y	$308.12	$471.96	$315.73	$471.96	$63.15	$126.29	$0.00	$63.15
		N	$308.12	$471.96						
	M	Y	$327.88	$471.96	$332.42	$471.96	$66.48	$132.97	$0.00	$66.48
		N	$327.88	$471.96						
05–14	F	Y	$79.33	$302.61	$99.94	$302.61	$19.99	$39.98	$0.00	$19.99
		N	$79.33	$302.61						
	M	Y	$75.18	$302.61	$93.54	$302.61	$18.71	$37.42	$0.00	$18.71
		N	$75.18	$302.61						
15–24	F	Y	$78.90	$291.50	$92.22	$291.50	$18.44	$36.89	$0.00	$18.44
		N	$36.09	$291.50						
	M	Y	$42.38	$291.50	$50.75	$291.50	$10.15	$20.30	$0.00	$10.15
		N	$20.79	$291.50						
25–44	F	Y	$72.61	$291.50	$81.04	$291.50	$16.21	$32.41	$0.00	$16.21
		N	$7.32	$291.50						
	M	Y	$43.16	$291.50	$52.38	$291.50	$10.48	$20.95	$0.00	$10.48
		N	$5.91	$291.50						
45–64	F	Y	$88.74	$319.27	$110.99	$319.27	$22.20	$44.40	$0.00	$22.20
		N	$12.22	$319.27						
	M	Y	$67.96	$319.27	$82.90	$319.27	$16.58	$33.16	$0.00	$16.58
		N	$9.57	$319.27						
65+	F	Y	$191.27	$342.40	$191.27	$342.40	$38.25	$76.51	$0.00	$38.25
		N	$191.27	$342.40						
	M	Y	$164.95	$342.40	$164.95	$342.40	$32.99	$65.98	$0.00	$32.99
		N	$164.95	$342.40						

Per capita management fees are paid irrespective of Access or Interim status, and are based upon PHO size:

• $9.61 per individual up to 20,000 and $4.67 per individual thereafter, for PHOs with fewer than 75,000 registered individuals

• $6.41 for the first 20,000, $5.83 for individuals 20,001 to 75000, and $5.25 for all others, for PHOs with more than 75,000 registered individuals

Definitions

PHO Types:

• Interim PHOs: more than 50% of the registered population of Maori or Pacific Island Ethnicity, or living in areas determined to be in NZ Deprivation Index deciles 9 or 10.

• Access PHOs: the remainder

Individual Characteristics:

• HUHC: High User Health Card – individual with 12 or more GP consultations in 12 months

• CSC: Community Services Card – identifies low income or beneficiary status of registered individual – irrelevant for registered patients of Access practices

Capitation Subsidy Type:

• GMS/Nurse: subsidy for first contact services provided by General Practitioner or practice nurse – nominally based upon an effective consultation subsidy of $36.40 for children under 6 and $26 for all other population groups eligible for low or reduced cost access, thereby presuming 13 fully subsidised visits for a HUHC young child and 8.5 for others; 6.3 partially subsidised visits per annum for a 65+ man and 7.4 for a 65+ woman (3.3 and 3.8 fully subsidised visits respectively assuming a $50 cost per visit).

• Services to Improve Access: capitation to develop access initiatives for high-needs populations (paid in addition to GMS/Nurse capitation

The structural instrument is community-based nonprofit Primary Health Organisations (PHOs), created to register eligible individuals, receive associated capitation monies, co-ordinate delivery of care to registered individuals and manage the associated service delivery contracts. PHOs are also charged with responding to the health needs and preferences of their communities and developing innovative ways of providing services that people can afford [4]. There are no restrictions placed upon the nature (either for-profit or nonprofit) of the entities with whom the PHOs can enter into contracts, or the nature of the financial or operational risk-sharing that these contracts may entail. The only requirement is that PHOs be nonprofit entities openly accountable to the public for their decision-making, with contracted service providers and communities being represented in their governance and decision-making [5]. As District Health Boards (DHBs) oversee the contracts with PHOs, the NZPHCS requires that PHOs are formed within DHB boundaries.

**Figure 1:**
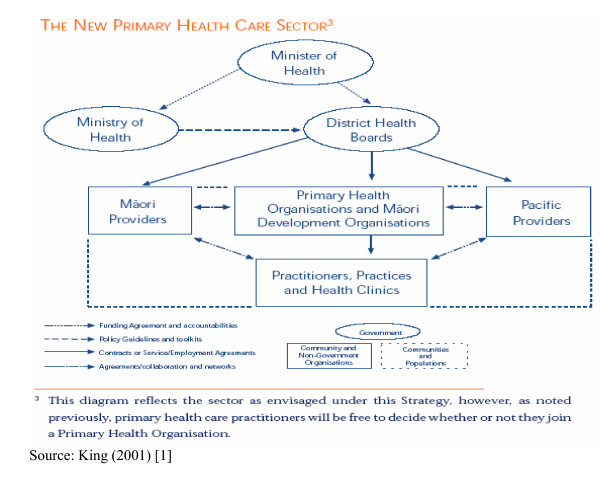
The New Zealand Primary Health Care Sector.

The relationships between the government, its primary policy-making agency the Ministry of Health, the 21 geographically-determined government service purchasing and delivery agencies the DHBs, the PHOs, service providers and patients under the NZPHCS is illustrated in Figure 1.

This paper examines the NZPHCS by analysing the changes in the contractual relationships and institutions relative to the pre-NZPHCS contracts and institutions. Specifically, it examines the intertwined implications of changing the principal government-funding instrument from a fee-for-service payment to a capitation payment, and the substitution of PHOs for service providers as the 'other party' with taxpayers, patients and the government in the social contract for overseeing the purchase and delivery of subsidised health care.

## Analysis of Contractual Changes

The change in the subsidy from a targeted welfare benefit upon the consumption of services to a universal subsidy independent of consumption alters the nature of the residual risk-bearing for variations in individual patient consumption of health services (that is, variations in individuals' underlying health states). Pre-NZPHCS, the government underwrote the risks of demand variation for a small number of subsidised individuals whilst the majority of individuals self-insured. Risk-sharing was required only for the subsidised proportion of the population. Under the NZPHCS, as over ninety percent of the population receives subsidies, the risks and costs associated with variations in the health states of the nearly the entire population are shared. In the first instance, PHOs are required to underwrite these risks, but can subcontract these risks onto service providers via service provision contracts. Via their ability to levy patient charges in addition to revenues received from PHOs, service providers may subcontract these risks further onto those who consume primary health care. Whilst the motivation for changes in locus of risk-bearing is to alter the behaviour of sector participants (principally service providers), the contracts that are emerging under the NZPHCS reveal that a reallocation of some of these risks is occurring in ways that are likely to lead to higher costs in total, an inequitable allocation of these additional costs amongst patients, and less efficient outcomes relative to the pre-NZPHCS contracts. The evolution of the entities engaging in contracting and the less-efficient contracts that they are entering into, appears to result from the acceptance under the NZPHCS that the pre-NZPHCS contractual arrangement allowing service providers to levy patient charges directly will continue under the new system. Whilst the strategy anticipates that structures and contracts will evolve over time in response to competitive pressures [6], as long as the practitioner right to levy patient charges independent of contractual constraints applied by the PHOs prevails, service providers continue to predominate in the governance of PHOs, and competitive pressures upon PHO-service provider co-operatives remains weak, it is likely that the current higher-cost arrangements will become entrenched.

### Pre-NZPHCS Arrangements

The NZPHCS arrangements mark a fundamental change to the public-private partnership between government and privately-owned service providers (principally general practitioners) that characterised government subsidy of primary health care in New Zealand between the late 1930s and 2002. The public-private partnership arose out of a compromise between medical practitioners and the government in order to allow the Social Security Act 1938 and the subsequent Social Security Amendment Act 1941 to be passed [7]. Whilst the government at the time wished to offer 'free to the patient' taxation-funded health care, as in England's NHS, there was widespread opposition from members of the medical profession, who wished to retain their autonomy as private sector owners of the businesses delivering medical treatment. The outcome of the compromise was a bifurcation of the health sector into fully government-owned and funded public hospital, maternity and mental health services and privately-owned primary and specialist services. The government agreed to partially fund services provided by the private sector, whilst private practitioners retained the right to levy additional charges to patients [8].

**Figure 2:**
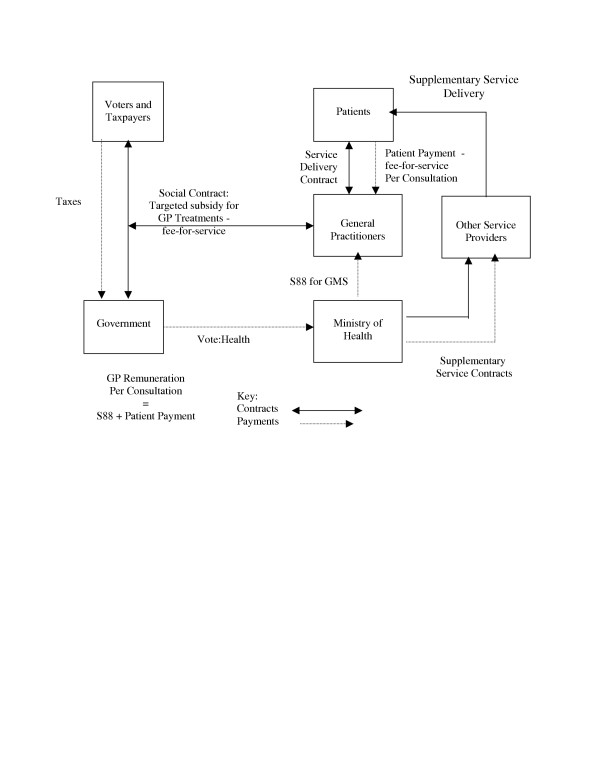
Pre NZPHCS Primary Health Care Contracts.

Under the arrangements brokered in the 1930s, the 'social contract' resulted in private sector general medical practitioners being paid a fixed fee from taxation revenue for each treatment provided to eligible patients (termed a 'Section 88 Payment for General Medical Services', after the relevant section of the Act in which it was instituted). The payments were 'fee-for-service' in that they were paid for each consultation rendered to eligible patients by eligible practitioners. Whilst for administrative simplicity the payments were made direct to the servicing practitioner, in effect they constituted a 'welfare benefit' granted to an eligible patient, in that they were a part-payment of the debt incurred by the patient to the practitioner when medical treatment was sought by, and delivered to, the patient. The level of the subsidy was determined by individual patient characteristics and paid upon consumption of services. General practitioners were free to levy patients for the difference between the 'Section 88' payment (subsidy) and their actual costs of service delivery – the balance of the patient's debt, which was termed the 'patient co-payment'. As all registered general practitioners were eligible to receive 'Section 88' payments, there were no constraints placed upon patient choice of practitioner. These contractual arrangements are illustrated in Figure 2. Initially, the primary health care 'welfare benefit' was universal, in that all citizens were eligible to receive financial assistance, although the amount paid varied with patient characteristics (principally age). In the early 1990s, the subsidies became more tightly targeted, with family income, patient age and patient health state (principally the number of visits made in a twelve-month period) being the primary determinants of both eligibility for, and the size of, the subsidy.

Over time, the 'welfare benefit' share of the cost of general practitioner consultations fell substantially. When the NZPHCS was implemented, the government's contribution amounted to only 30% of general practitioner revenues [9]. The remaining 70% came from out-of-pocket expenses, or patient-funded private insurance contributions. Historically, private medical insurance has provided only a very small share of New Zealand primary health care costs, with fewer than 35% of the population having private medical insurance [10], and only 15% of total private medical insurance expenditure being applied to primary health care costs in the late 1990s [11]. As the range of available primary health care treatments increased, dissatisfaction grew with the restriction of subsidy payments solely to general practitioners and the 'medical intervention model' that it incentivised, potentially at the expense of models and practitioner types (e.g. nurses, dieticians, physiotherapists, social workers, educators) promoting the pursuit of wellness in the population.

During the 1990s, additional government funding was applied to a variety of contracts with a wider range of multi-disciplinary primary health providers (often, but not exclusively, nonprofit charitable trusts), typically serving high-need individuals or communities (often communities of specific geography, ethnicity, disease state or high health need). These contracts were typically either capitation-based (a fixed annual fee per individual eligible to receive the services offered) or price-and-volume-based (a fixed fee for a specific number of consultations). These relationships are also illustrated in Figure 2. In addition, a number of general practitioners moved voluntarily onto capitation-based payments as allowed under the Social Security Amendment Act 1941. Capitated practitioners retained the right to levy patients for the shortfall between capitation subsidies and actual costs. It is estimated that as many as 22% of general practitioners were receiving capitation funding in 2001 [12], although the number of patients involved is unknown. By 2001, government funded around 40% of the costs of all primary health care via both 'Section 88' and other contractual arrangements [13].

### NZPHCS Arrangements

The NZPHCS fundamentally rewrites the 'social contract' between taxpayers, the government and general practitioners, with consequent changes in responsibilities and cash flows in the sector. The private-sector partners who receive government funding for primary health care under the new 'social contract' are no longer general practitioners and other service providers, but are the newly-created nonprofit Primary Health Organisations (PHOs). PHOs are charged with recruiting and registering patients for whom they will be responsible for purchasing and co-ordinating a range of primary health care services that will deliver upon the NZPHCS objectives of improved health outcomes and reduced disparities. PHOs are free to enter into contracts with any service providers (including, but not restricted to, general practitioners and providers receiving government contracts under the 1990s arrangements). Service providers who previously participated in 'Section 88' or other government-funded primary health care contracts must now enter into individual agreements with PHOs if they wish to receive income originating from government sources. Service providers are now no longer directly parties to the NZPHCS 'social contract': they participate in it only as subcontractors to a PHO. The terms and conditions of PHO-provider contracts appear to be freely negotiable between the parties concerned. In principle, this freedom of contract implies that PHOs need not bound by the requirements of the historic 'social contract' in their dealings with general practitioners or any other service providers.

Citizens who wish to receive government subsidies for primary health care under the NZPHCS 'social contract' are now required to have an explicit contractual relationship with a PHO. Unlike the pre-NZPHCS system, subsidies are universal, and are paid every quarter, irrespective of the actual quantity of health care a PHO-registered individual actually consumes in that period. Whilst for administrative convenience government payments are made direct to the PHO, it is the patient's contract with the PHO that determines government-sourced cash flows. In effect, the subsidy is a notional 'voucher' allocated to an individual and paid to the individual's chosen PHO upon production of evidence of the existence of a patient-PHO contractual relationship. A new contractual relationship, distinct from any other relationship that may exist between the individual as a patient and a service provider who may be acting as a PHO, is required because the basis of government funding has changed from the historic 'Section 88' instances of treatment (information historically supplied by service providers) to instances of PHO registration (a metric independent of any consumption of services and hence independent of service providers).

The patient's contractual relationship with the PHO grants the PHO both the right and the obligation to enter into contracts with service providers on behalf of the registered patient. The PHO-contracted service provider's obligation to provide subsidised care for a PHO-registered patient, either when the patient falls ill and requires treatment, or in respect of any well-patient services the PHO-provider contracts specify (e.g. preventative medicine, education), stems from the patient-PHO-service provider contractual nexus rather than from any other obligations or arrangements that may exist or may have existed historically between the patient and the service provider. Whilst capitated patients may nominate a preferred primary care provider, they are not restricted from seeking care from another provider. The NZPHCS allows for cash clawbacks by PHOs/practitioners delivering treatment for services provided to patients registered by other PHOs/practitioners. Clawbacks are made on a per-treatment basis using population-based averages of the number of visits made by patients of the relevant patient's class, and the quarterly capitation payment for that class of patient. Clawbacks can amount for up to 10% of quarterly general practitioner revenues [14].

**Figure 3:**
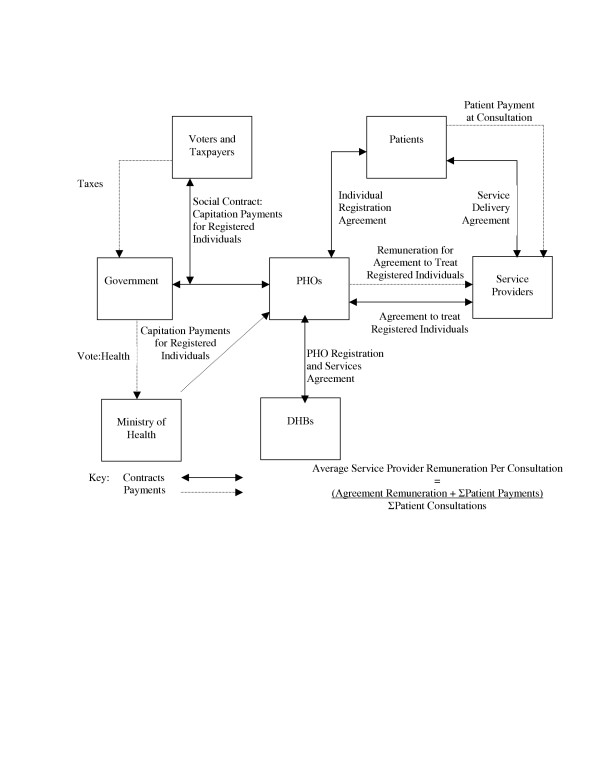
NZPHCS Primary Health Care Contracts.

The new contractual relationships outlined in the NZPHCS are illustrated in Figure 3.

### Transition Between Strategies

Whilst Figure 3 shows a direct relationship between the patient and the PHO, in practice there is rarely a direct, independent relationship. For administrative convenience, contracted service providers have been utilised as agents of the PHOs to facilitate the creation and maintenance of patient-PHO relationships: "existing lists of the patients who normally attend a practice or health clinic may form the starting point for enrolment" [15]. Indeed, a patient 'signals' a change in the PHO-patient relationship in most instances not on the basis of any direct transaction with the PHO, but by electing to change the preferred primary service provider. If the second provider has a contractual relationship with a different PHO from the first provider, the patient is deemed to have 'changed PHOs'. PHO revenue streams (and by extension, participation by their service provider-agents in government-subsidised service delivery contracts) are thus determined by the relationships between patients and the provider-agents. As PHO formation requires certainty of current and future revenue streams, and this certainty relies upon ongoing provider-patient relationships, most PHOs have been created based upon existing networks of service providers, such as general practitioner-governed Independent Practitioner Associations and community health organisations (including those based around communities of specific ethnicity) with strong provider networks.

The PHO creation arrangement also leads to PHOs binding service providers to exclusive contracts, denying them the right to enter into contracts simultaneously with any other PHO. Exclusive contracts are necessary as service providers with multiple PHO contracts cannot be relied upon to recruit and retain patients for a specific PHO. Consequently, PHOs compete with each other in order to sign up service providers rather than competing with each other to sign up patients. The PHO-service provider relationship 'crowds out' any incentives for PHOs to develop and manage direct relationships with patients independent of service providers, and results in effective competition in the sector being restricted to competing vertical alliances of PHOs and their exclusive providers [16]. The NZPHCS further requires that a PHO's contracted service providers (both those involved it its creation, and those who join subsequently) be active participants in the organisation's ongoing governance and management: "all providers and practitioners must be involved in the organisation's decision-making" [17]. Existing service providers are thus pivotal to the creation and operation of PHOs, with existing collectives advantaged by utilising existing relationships to 'become a PHO'. The operation of PHOs formed on this basis is likely to be strongly influenced by the providers around which the PHOs are formed.

The actual PHO registration, governance and management processes that have emerged raise the question of whether under the NZPHCS service providers are operating as contracted agents of PHOs, or whether PHOs organised around existing provider groups are in effect acting as service provider co-operatives (or even 'provider agents'). If the latter is an accurate representation of actual behaviour, then PHOs offer those providers (or groups of providers) who received subsidies pre-NZPHCS and who, through their collective bargaining ability, historically exhibited significant ability to influence the terms and conditions upon which government primary health care spending was applied, the ongoing ability to ensure access to, and to determine the contractual application of, government subsidies. If service providers control both PHO decision-making and PHO patient registration, it is unlikely that PHOs can freely enter into service provider contracts that are optimal for registered patients and contracting DHBs, and in the interests of the long-term financial viability and independence of the PHO itself, without jeopardising the relationships with providers upon which they are dependent for deriving their membership and hence their current and future revenue streams. If significant impediments to PHOs' independence and freedom to contract exist, then there may be substantial obstacles to the NZPHCS achieving its stated objectives efficiently using the PHO instrument as it is currently defined.

#### Registration Incentives

Outside of the NZPHCS, patients and service providers have complete freedom to enter into any contractual relationship of their choosing. The historic 'Section 88' payments will prevail at the 2001 levels. However, patients who opt for the pre-NZPHCS arrangements (and the PHOs who would otherwise register them) are denied access to the substantially more generous subsidies available under the NZPHCS. For example, a patient not qualifying for a 'Section 88' subsidy who consumes no services will generate revenue but no cost for a PHO under the NZPHCS. The same patient receives no funding at all if opting to remain under the 'Section 88' payment system, even if services are consumed, as there is no subsidy eligibility under this regime.

The availability of subsidies unrelated to actual consumption of health care is intended to provide strong incentives for PHOs to register individuals, consistent with the strategy's intention to increase the quantum of care provided to individuals who have not historically consumed health care and have poor health states. However, the PHO incentive to register is strongest in respect of those individuals who historically have not consumed large quantities of care due to their better-than-average underlying health states. These individuals bring revenues to the PHO based upon population averages, but the costs they incur are less, as they will likely consume fewer services. This exposes the NZPHCS to the risk of PHOs and their registering agents engaging in active patient pool selection ('cream-skimming') as it favours registering entities that can identify and register more profitable healthier-than-average individuals and exclude individuals who are less healthy than average and therefore less profitable. Yet it is the less-healthy-than-average individuals whose under-consumption of services under the pre-NZPHCS system was perceived to be contributing to substantial differences in patient health outcomes. Such pool selection incentives were not present under the 'Section 88' fee-for-service contracts, as service providers were fully compensated for all treatments provided, paid either by the individual or a combination of the individual and the state subsidy only in respect of treatments provided, irrespective of the patient's underlying health state.

### The NZPHCS in Action

By December 2004, under the NZPHCS, capitation subsidies were being paid on behalf of 3.7 million New Zealanders (92.5% of the population) to 77 nonprofit PHOs [18], who enter into contracts with service providers for the delivery of primary health care to those individuals who fall ill. The subsidies vary depending upon the age, gender, income and historical health state of the registered individual, and upon PHO characteristics, determined by registered population size, and the ethnicity and deprivation levels of the registered population (see Table 1). Two types of PHO exist based upon these characteristics: Access and Interim. Higher-subsidised Access PHOs are required to have a registered patient base with at least 50% of individuals exhibiting specific ethnicity and financial deprivation characteristics [19].

General practitioners have taken a leading role in PHO formation. For example, thirteen of the South Island's seventeen PHOs are affiliated to Southlink IPA, and fourteenth, New Zealand's largest, is also based around an existing general practitioner-based primary health care collective. Consequently, over 92% of South Island PHO registrants belong to a general practitioner-formed PHO [20]. Practitioner influence over PHOs need not be confined to practitioner involvement in PHO formation. Wellington Independent Practice Association Limited (WIPA Ltd), a private company owned by fifty-nine general practitioners, has contracts to provide all management services to five PHOs across three DHBs in the lower North Island. The WIPA-managed PHOs have a combined market share of over 85%, and their contracts grant WIPA Ltd rights to act as if it were the PHO in respect of strategy, financial operation, service contracting and service development [21]. Nationwide, around 90% of the registered population is served by PHOs formed around general practitioner-dominated alliances.

The 77 PHOs in existence at December 2004 range in size between 3300 and 330,000 registered patients. They are also unevenly spread both geographically and by funding type. Higher-subsidised Access PHOs have on average 19,000 registered patients (median 11,200), whilst lower-subsidised Interim PHOs average 53,000 patients (median 31,200) [22]. South Island DHBs have on average 3.2 PHOs in their territories, whilst North Island DHBs have an average of 4.6 [23]. Only six percent South Island PHOs qualify for higher-subsidised Access funding, compared to sixty percent of North Island PHOs. Over 41% of individuals registered in the higher-subsidised Access PHOs in 2003 did not exhibit the population-based characteristics upon which the subsidy differentials are based [24]. If the population-based demographic characteristics upon which the subsidy differentials are based are good proxies for the actual costs of the differing underlying health needs of individuals, the high registration of non-Access individuals in Access PHOs suggests that active pool selection is occurring based upon subsidy differences.

Competition between the 77 PHOs is negligible, largely because of the geographic constraints that they be formed within DHB jurisdictions. Using as a benchmark for the presence of competition a three-firm concentration level of PHOs in each DHB area of less than 70%, and the largest PHO having a market share (measured as a PHO's share of the total PHO-registered population in a DHB area) of no more than 40%, there is little evidence of any meaningful competition occurring between PHOs [25]. On closer examination, most PHOs enjoy a local geographic monopoly. Even where patients have a notional choice of PHOs, effective choice does not appear to exist as PHOs are differentiated principally upon ethnicity and subsidy types, rendering the ability for the majority of patients to substitute practically non-existent. Higher-funded Access PHOs have no incentive to register individuals with non-Access characteristics if doing so would threaten the ability to claim higher subsidies for the entire registered base. Most Access PHOS are already close to the thresholds for non-Access registrants, making such substitutions unlikely to occur, even if there were no implicit ethnicity barriers discouraging substitution [26].

## Discussion

The evidence to date suggests that despite the intention of the NZPHCS to change the identity of the 'other party' to the 'social contract' by establishing PHOs as new contracting entities, existing service providers, and in particular, general practitioners, have utilised the NZPHCS structures to replicate what appears to be similar relationships and responsibilities to those prevailing pre-NZPHCS. If general practitioners can dominate PHO decision-making, then under the guise of PHO governors they can continue to act as the de facto 'other party' to the social contract, as detailed in Figure 2. But whilst the NZPHCS structures may enable them to take these roles and in doing so preserve the outward form of previous relationships, the change in the funding instrument from targeted fee-for-service welfare benefits to universal insurance premium subsidies fundamentally alters the nature of the contracts which these relationships must fulfil. If the relationships prevail under the assumptions of the pre-NZPHCS funding contracts, but are required to serve the objectives intended under the NZPHCS arrangements, the potential exists for significant divergence between the intentions of the strategy and its outcomes. Intended outcomes may not be achievable, or if they are, it may be at substantially greater cost than if the NZPHCS relationships and contracts were built afresh. The balance of the paper discusses the key changes to contracts and relationships arising from the creation of a universal insurance instrument, and analyses the ways in which the relationships that have emerged under the NZPHCS institutions based upon pre-existing relationships are likely to affect the ability to deliver the intended objectives efficiently.

### Subsidised Health Care: a Two-Sided Market

A fundamental distinction between markets for health care and markets for many other products is that patient-based demand for health care is a 'derived demand' arising from the occurrence of a stochastic event – illness. The unpredictability of falling ill, and the often-substantial costs associated with seeking treatment, creates demand uncertainty. In order to manage this uncertainty, individuals may prefer to 'pool' some of their risks and the associated costs of falling ill via private sector insurance schemes or centralised, taxpayer-funded institutions. These centralised entities then enter into contracts with service providers on behalf of the individuals to ensure both that treatments are provided when the patient falls ill, and that the service providers will receive payments for their services [27]. The central contracting entity, which may be an insurance company, a government agency or some other institution, becomes the 'hub' in a 'two-sided market' [28], entering into contracts on the one side with the individuals who seek certainty of access to and payment (or at least part-payment) for treatment, and on the other side with services providers who will deliver those treatments. The 'hub' entities maximise their profits (or optimise the health purchasing for their registered populations) by pooling the revenues received in respect of their registered populations and purchasing services only in respect of that subset of the pool that suffers an 'adverse event' (falls ill) and demands services. The two-sided market thus comprises a risk management (insurance) market on the one side, and a health service purchasing market on the other.

#### The Pre-NZPHCS 'Two-Sided Market'

Prior to the NZPHCS, government via the Ministry of Health operated the 'primary health care hub' in respect of the small proportion of individuals targeted for subsidised primary health care. Revenues in respect of targeted individuals were provided from taxation, comprising the 'risk management' component of the two-sided market, whilst the 'Section 88' agreement and payments, and agreements with other service providers, comprised the 'service purchasing' component. The government underwrote ('insured') the costs of any variations in the health states (and hence demands for health care) of the targeted individuals. The sicker they were, and the more treatments they sought, the higher the government's costs. Payments came from a single pool provided by all New Zealand taxpayers, and were made to providers when targeted individuals consumed care. The 'welfare benefit' payment was an output of the insurance 'hub' as it was a payment for services purchased in the service purchasing market.

As the majority of citizens were not eligible for targeted welfare benefits for primary health care, they had no need for the services of the taxpayer-funded 'primary health care insurance hub'. They were fully responsible for all of their costs of treatment. Costs from any variations in these individuals' health states were borne out-of-pocket, either directly in payments for services, or via private health insurance. These individuals were said to be 'self-insuring'. These patients paid the full costs of their treatment direct to service providers.

Under the fee-for-service payment arrangements pre-NZPHCS, service providers bore none of the insurance-related financial risks associated with variations in the health states of any of their patients, either self-insuring or targeted welfare beneficiaries. Each instance of treatment was fully paid for, either wholly by the patient, or by a combination of patient payment and taxpayer subsidy. Whilst some practitioners voluntarily waived charges for some payments, this is a normal commercial decision made by any business operator who chooses to offer selected discounts to specific clients, and does not impose any insurance-based risk management obligation in respect of that client, as is the case in the two-sided insurance market. Providers' engagement in the two-sided market was thus solely as suppliers of services.

As in the case of any subsidy where the patient does not pay the full cost of treatment out-of-pocket, subsidised patients will likely consume more care than is necessary (e.g. over-consumption by the 'worried well' and supplier-induced demand, where providers, knowing that the patient is not paying the full costs, recommends more expensive treatments than necessary or treats beyond the point where a 'cure' has been effected – known as moral hazard behaviour [29]). As only a small proportion of individuals received subsidies pre-NZPHCS (and most paid some form of co-payment out-of-pocket), moral hazard costs were likely to be relatively small. Any moral hazard costs that were incurred by subsidised patients were borne collectively by the entire pool of New Zealand taxpayers. Furthermore, as there was only one state-funded insurance 'hub' pooling risks for targeted individuals, and participation by eligible individuals in the pool could not be denied, the incentives that typically face competing insurers to manipulate the risk profile of the patient pool by 'cream-skimming' were absent.

#### Two-Sided Markets Under the NZPHCS

At the simplest level, the NZPHCS fundamentally changes both the size and the locus of residual risk bearing in respect of health state variations in the recipients of subsidised health care. Multiple PHOs, with the potential to compete against each other, rather than the single Ministry of Health, become the 'hubs' in the two-sided primary health care market, and all citizens who register with PHOs become parties to the insurance-based health-state risk pooling and service purchasing that the PHOs, rather than the Ministry of Health, are charged with undertaking. Government subsidies change from being an output of an insurance hub (i.e. service purchasing payments) to an input into one (i.e. insurance premiums). The structures and financing arrangements espoused in the NZPHCS model appear consistent with the trends emerging in many countries, where with the use of premium subsidies "government plays a major role in assuring that insurance coverage is universal and affordable, but with competition in the provision of insurance and of medical care, in order to stimulate efficiency and provider responsiveness to consumer preferences" [30]. Whilst questions have been raised about the extent of meaningful competition present in New Zealand between PHO-insurers, the fundamental shift in direction under the NZPHCS to a multi-insurer model nonetheless invokes all of the cost consequences that are associated with risk management under any insurance-based scheme.

#### Insurance Consequences: Universal Insurance and PHO-Insurers

Firstly, as all citizens who register with a PHO become eligible for subsidies, no registered citizen will face the full cost of health care treatments provided. Whilst increased subsidies and extensions from targeted to universal eligibility are intended to induce those who could not afford to pay for care previously to now seek it, these instruments will also lead to a rise in the levels of inefficient over-consumption relative to the pre-NZPHCS counterfactual as the entire population now faces the incentive to inefficiently over-consume. Such behaviour has been documented previously in New Zealand in 1996 in respect of targeted subsidies for under-six-year-olds becoming universal [31]. Furthermore, the new subsidies will 'crowd out' at least part of the private contributions that the newly-subsidised individuals would have previously paid. Total costs of the system will rise, in many cases for no additional health gain.

Secondly, although the quantum of government funding for primary health care has been increased, the change in the nature of funding away from fee-for-service to capitation shifts the costs of demand variation in subsidised patients' health states away from government into the private sector. The PHOs have become the insurance hubs. Capitation payments become insurance premium subsidies, and PHOs now bear the risks of patient demand variation. Once the capitation levels are set, the government faces a predetermined charge every quarter, independent of variations in the demand for health services based upon actual patient health states. The only variations government faces attend to the number of individuals for whom subsidies are paid and any movement of individuals between subsidy classes. In the first instance, responsibility for cost variations due to variations in the health states of both those individuals that the government used to insure, and a very much larger number of additional individuals who previously self-insured but are now party to risk-sharing agreements, has been placed upon the PHOs. However, where and how the costs of these variations are ultimately borne (and any surpluses that might accrue), and their sizes, will depend upon the decisions made by PHOs. Specifically, the ultimate size and allocation of costs and risks associated with patient demand variations will be determined by the contracts PHOs have with their service providers and their registered populations.

#### Relationships and Residual Risk-Bearing

The change in the funding instrument of the 'social contract' changes the relationships between all participants in the sector. PHOs become both health insurers and health service purchasers for their registered populations. Government is now simply a supplier of funding to the insurers and managers of service delivery contracts, rather than being the insurer and manager of those contracts itself. The subsidy is a partial contribution towards the insurance premiums of registered individuals. Government's sole role is to specify the size of the subsidies, and any contractual obligations associated with their application (e.g. minimum service ranges and qualities, reporting requirements). Patient out-of-pocket payments are now not a part- or full-payment of the costs of a service rendered by a service provider (a 'co-payment' in the classic fee-for-service subsidy environment), but a premium 'top-up' that brings the government premium subsidy up to the level of the actuarially-determined premiums that will be required to resource the PHO's contractual purchases of treatments. Even though for administrative convenience service providers collect patient payments, patient payment size will be determined ultimately by the costs of managing the insurance pool for, and of delivering treatments to, the PHO's entire registered patient pool. Patient payments must necessarily include factors that reflect the costs and risks of health state variations in the treatment-seeking behaviour of the PHO's entire registered patient base, and the risk management practices of the PHO and its subcontracted providers. These costs are incurred in addition to any shortfall between any government subsidy paid and the suppliers' actual costs of providing any specific contracted treatments.

The change in insurance responsibility means that any increase in moral hazard behaviour of patients alone makes it extremely unlikely that any increase in average subsidy under the NZPHCS will lead directly to a dollar-for-dollar decrease in the average patient 'co-payment' charged by providers, as it did under changes to the fee-for-service 'Section 88' subsidies. This occurs simply because the increases in cost from moral hazard actions previously borne collectively by the government and the taxpayer under Vote:Health are now reflected directly in the prices paid by patients, as it is the PHOs and their subcontractors who must now underwrite these risks. As long as the solitary instrument allowed under the NZPHCS for collecting any revenue shortfalls from demand variation is the patient payment to service providers, these payments must necessarily include a component to meet such costs. Thus it is not surprising that the Minister of Health has found that average patient co-payments in the "6–17 age group could not be seen to adequately reflect the increase in funding for that group in October 2003" [32] – indeed any other result would suggest an atypical consumer response to an increase in subsidy.

### PHOs as Managed Care Organisations

As the NZPHCS institutions and relationships require PHOs to act as both insurers and service purchasers, it may be inferred that PHOs are acting as 'managed care organisations', balancing the costs of and demands for, primary health care within defined budgets (set by capitation subsidies or premiums) by matching the allocation of services purchased to the needs of the enrolled population. Managed care models have evolved around a set of fiscal and practice-based strategies, largely in response to the over-consumption of care, overly-high costs and weak incentives for providers to constrain costs that attend fee-for-service-subsidised health care systems [33,34]. Practice-based managed care strategies seek to "reduce variability in medical care by identifying 'best practices' and promoting adherence to guideline-based decision making. This includes evaluating the appropriateness of services rendered and the level of care necessary to provide the services" [35]. Practice-based managed care strategies appear appropriate for addressing the specific NZPHCS objectives of co-ordinating care across a range of providers, responding to community needs and continuously improving quality by better co-ordination, service innovation and use of information. Fiscal strategies in managed care typically involve some degree of financial risk-sharing between service purchasers and service providers, based upon the premise that it is more efficient for at least some of the risk associated with patient demand variations typically underwritten by insurers to be "borne by the individual physician for whom it is not a risk, but a controllable cost" [36]. Capitation payment for service delivery is one such fiscal strategy, along with preferred provider networks, price-and-volume contracts and other financial restrictions upon the freedom of practitioners to supply and commission treatments [37].

#### Managed Care Fiscal Strategies to Alter Provider Behaviour

Capitation payments for remuneration of service delivery are not axiomatic under the NZPHCS simply as a consequence of the insurance premium subsidy inputs paid to PHO-insurers being capitation payments. Any use of capitation payments by PHOs in respect of their service delivery contracts occur on the 'output' side of the 'insurance hub', and should be assessed in respect of their efficacy in assisting the managed care entity in achieving its balancing of demands and costs of care within its budgets. Each period, the insurer must pool the funds received in respect of all registered/insured individuals – the premiums – and from that income pay the costs of treatment for which the insurer is liable for that subset of the registered/insured population that requires services. The insurer thus balances the costs incurred by individuals against the revenues received in respect of the total registered base, and assumes responsibility for funding any shortfall (or keeps as a profit any surplus) that arises if the costs incurred are higher than the averages upon which the revenue is based because the registered base is less healthy than the average and consumes more services than average (that arises if the costs incurred are lower than revenue averages due to a more healthy than average registered base that consumes fewer services than the average). The insurer thus bears the financial variations associated with any variations in the patient health state from the averages upon which revenue is determined. Managed care entities are thus incentivised to look for ways to reduce costs in order to increase profits (or increase the quantum or quality of care provided within its fixed income budget). Even nonprofit managed care entities are incentivised to pursue higher profits, as the higher the profits (lower the costs) the more benefits the organisation is able to deliver to its beneficiaries, and the 'better' the nonprofit entity is deemed to perform [38].

Managed care entities can reduce the total cost of health care by using terms in the service contract to incentivise service providers to restrict their inefficient cost-causing behaviour. The contractual terms 'share' the managed care organisation's risks (and the associated costs) of variations in patient health states, and therefore demands for service, with the service providers. If providers can reduce costs (e.g. with preventative interventions and education), then financial risk-sharing will incentivise providers to alter their treatments in cost-conscious ways as they have to bear some of the costs associated with variations in patient health state, in the same manner as the insurance company. Financial risk-sharing contracts incentivise providers to reduce their costs by separating at least some of the determinants of providers' income streams (e.g. using capitation payments and discounted-price-and-volume payments) from the determinant of their costs (typically instances of care delivered to patients). With some of their revenues now 'fixed', providers can maintain their previous levels of profitability only by paying more attention to constraining their costs [39]. However, whilst provider costs can be constrained by reducing the extent of inefficient supplier-induced demand and other wasteful expenditure, and reducing the quality of service provided to an 'efficient' level, providers may also respond by reducing the quality provided below the 'efficient' level (e.g. overly-short consultations, queueing, rationing), further sharing the risks and costs with other parties if feasible (e.g. passing costs onto other contracting parties, shifting demand to other service providers), or actively engaging in 'selecting' a patient base that is lower-cost on average than the level at which the provider is remunerated, and thereby reducing the expected costs of the firm.

#### Managed Care Contracts and Incentive Intensity

The challenge for managed care organisations is to determine how much of the demand variation it is reasonable to share, given that not all cost-causing events are controllable by practitioners (e.g. a practitioner cannot influence the genetic predetermination or environmental circumstances that increase the health risk and hence demand of some individuals). If too much risk is shared, providers will pursue undesirable cost reduction activities at the expense of other objectives [40]; if too little is shared, then the costs of health care to the managed care insurer (and by extension the providers of its revenues) will be higher than necessary. Capitation funding provides the strongest cost-constraint incentives of all the fiscal strategies upon providers, with the strength of the incentive increasing with the proportion of revenue received from capitation [41]. Significant changes in practitioner behaviour have been observed in the United States under contracts with very low-powered financial incentives [42], suggesting that schemes with very high levels of capitation may over-incentivise provider cost-containment activities at the expense of other objectives, by placing more financial risk than is optimal upon providers [43].

Typically, managed care schemes also involve some compromises for patients. In order to constrain the prices paid by purchasers (via taxation, insurance premiums and out-of-pocket patient payments) in highly-subsidised systems and to ensure value-for-money spent, there must necessarily be some reductions in patient choice of practitioners, available treatments or treatment quality, and potentially even service rationing and queueing, relative to the counterfactual of an unrestrained fee-for-service payment regime [44]. Indeed, these are the very types of compromise that attend the provision of fully-capitated (via population-based budgets) state-funded public hospital services offered in New Zealand by the District Health Boards, which are in effect the 'care managers' in respect of these services for their designated geographic (equivalent to registered) populations, and the fully state-funded Primary Care Trusts in England's NHS [45]. Managed care schemes are therefore typically also associated with increased levels of overt monitoring relative to fee-for-service indemnity-type schemes in order to ensure that the service quality, range and availability do not fall below predetermined minimum acceptable levels [46]. Such monitoring adds substantial cost overheads to the managed care model, but as long as the cost reductions achieved exceed the additional costs within acceptable service qualities, managed care models can be more efficient than traditional indemnity-based fee-for-service insurance models [47].

### Provider Charging and Managed Care Fiscal Strategies

In order to effectively design and manage contracts with service providers, so that the service providers are given sufficient incentives to constrain costs but still pursue other objectives, and are unable to shift onto other entities the risk that the managed care organisation deems providers should optimally bear, the managed care organisation typically uses contract terms to control all aspects of service provider remuneration in respect of treatments provided to registered patients. All service provider revenues relating to the contracts are typically provided from managed care funds (from premium/subsidy income or co-payments from insured individuals). Where co-payments are made direct to the service provider, these are generally in the form of a fixed deductible (excess) at a level determined by the managed care organisation rather than a fee set by the service provider to recover costs.

Indeed, any financial incentive effect upon a service provider in a contract with a managed care organisation will be 'undone' if the provider has the arbitrary ability to set patient co-payments to recoup any costs not provided by the managed care contract remuneration. A provider that has power to set co-payments has the power to shift onto patients that proportion of the risk that the managed care entity has deemed the provider should optimally bear. Furthermore, any financial incentives imposed by the managed care entity to alter provider behaviour (e.g. to induce increases in preventative interventions) are also reduced. Neither the cost savings nor service improvements sought from the financial risk-sharing contract are likely to be delivered to the extent anticipated by the managed care organisation when designing the contract. If any behaviour changes are educed in such a contractual arrangement, they will result from the practice-based strategies of the managed care organisation rather than the fiscal strategies.

If a provider with a financial incentive contract has the ability to set patient co-payments independently of the contract, then the provider can replicate the cash flows and absence of patient demand variation risk-bearing that attend a fee-for-service payment scheme. The provider fee-setting ability thus renders futile any attempt by the managed care organisation to influence provider cost-causing behaviour using fiscal strategies. The patient ends up paying the costs of the provider's share of the risks directly, in addition to the costs of the premium paid to the managed care entity. Any low-cost benefits that managed care models promise relative to fee-for-service insurance schemes are therefore also negated. The patient/consumer pays the extra costs in addition to facing the restrictions in choice, provider availability and service quality that attend the managed care model. Where the patient can balance the costs of premium, co-payment and service restrictions against the costs and benefits of alternative heath insurance models, if the managed care package is unfavourably costly, the patient will purchase an alternative. Overly costly managed care models where providers can arbitrarily levy patient charges will be unable to survive in the face of competition from other, less costly models such as provider-constrained managed care organisations and fee-for-service indemnity insurance arrangements.

If, however, the patient does not pay both the premium and the co-payment (e.g. the premium is paid from taxation revenue rather than directly from patient funds, so that the patient cannot 'internalise' the trade-off between the size of the premium and the size of the co-payment), any patient-based comparison with an alternative service is based solely upon the size of the co-payment that the patient faces. The higher the level of the premium subsidy, and the smaller the amount of the premium subsidy that can be transferred to an alternative insurer-provider model, the less favourable any competing alternative will appear to the patient [48], and the less likely it is that substitution to other models will occur. If the market for health care provision lacks the competitive discipline provided by alternative models, the overly-costly insurer-provider combination will persist, even though the alternative combinations would be less costly in total, resulting in persistently higher costs of primary health care than would be achievable in an environment where alternative models can freely compete.

### PHO Fiscal Strategies and the 1938 Practitioner Charging 'Compromise'

The principal challenge facing New Zealand's managed care PHOs is that the very nature of the institutions and structures that have emerged under the NZPHCS pose some significant barriers to PHOs that restrict their ability to act as true managed care organisations. The stated intentions of the NZPHCS show contractual relationships between patients and PHOs (Figure 3), indicating that PHOs could charge registered individuals directly to recoup the difference between capitation subsidies and actuarially-calculated insurance premiums to fully-fund a managed care entity and utilise fiscal strategies to incentivise service providers to manage costs. In principle, such a funding arrangement would allow PHOs to develop a range of contractual relationships with a variety of providers that reflect both the extent of risk borne by each PHO given the health state of its patient base and the amount of risk desirable to be shared with specific providers in order to educe the desired behaviour changes, as in true managed care fiscal strategies, whilst simultaneously allowing patients to make the cost tradeoffs and select their desired PHO insurer on the basis of a bundle of cost and service characteristics. However, if the fundamental tenet of the 1938 compromise that allowed medical practitioners receiving income from government sources to levy additional charges on patients directly and independently of the PHO has been carried over into the NZPHCS, it renders impotent any attempt by PHOs to use fiscal strategies to constrain costs or incentivise desired provider behaviour.

#### Unconstrained Practitioner Charging Rights

Mandatory reporting by PHOs to their DHBs under the NZPHCS is based upon "the fees that their practices will be charging for standard consultations to the individuals in different groups" [49]. This suggests that practitioner charging of patients at the point of service consumption is the predominant, if not the only, way envisaged for PHOs and their subcontractors to recoup any additional costs of primary health care not covered by government subsidies. Even though alternative PHO charging models are theoretically feasible, 'grandfathering' of the historic right of medical practitioners to levy charges upon patients at service consumption has become the default method of premium top-up collection. Unless PHOs can contractually constrain the right of practitioners to charge patients, they will be able to operate as managed care entities only in respect of practice-based strategies.

There is little evidence of any constraints on general practitioners' rights to independently charge occurring in practice under the NZPHCS, at least in respect of the contracts between PHOs and the independent, for-profit private sector general practitioners that comprise the vast majority of the general practitioner workforce. The pro-forma 'back-to-back' contract offered on the Ministry of Health website as a model contract between general practitioners and PHOs (and is therefore likely to be similar to that between all private general practitioners and their PHOs) presumes that the PHO will simply 'pass on' capitation payments related to service provision directly to the service providers in respect of those patients that the service provider 'registers' with the PHO, suggesting that no such contractual constraint is being effected by the majority of PHOs. Whilst charged with managing risks as an insurance company on the one hand as a consequence of the shifting of responsibility for demand variation from the previous insurers (government and self-insuring individuals), PHOs are unable, due to the 'grandfathered' expectation of practitioners that they can still arbitrarily charge patients, from engaging effectively in using fiscal strategies to manage the financial risks that normally attend insurance-based managed care organisations.

#### Governance Arrangements and Contractual Content

Furthermore, as long as service providers dominate the governance of PHOs, it is extremely unlikely that PHOs will enter into contracts with their contractor-governors that share risks optimally by constraining the individual practice behaviour and profit-making potential of those selfsame contractor-governors. If the PHO cannot actively manage its financial risks using contracts, its optimal strategy is not to bear any financial risks at all. Thus, the default PHO contracting stance is likely to be one that simply shifts the capitation payments to service providers, who can then recoup any additional costs directly from patients.

As patients face barriers to exiting the subsidised system (they cannot take their state subsidies with them to a private insurer or any other practitioner operating outside the NZPHCS arrangements), and competition between PHOs is effectively non-existent, there are few competitive pressures acting to discourage PHOs from 'passing on' financial risks, or to constrain any additional costs that will arise from these actions. It is therefore extremely unlikely in the New Zealand context that capitation payment of service providers will be used in an actuarially-reasoned way to share a defined proportion of manageable risks between PHOs and service providers in order to exert controls upon service provider behaviour. Rather, the risk exists that as premium subsidies from government increase, and these are passed indiscriminately onto service providers, service providers may face overly-strong financial incentives. Whereas under constrained systems, these overly strong incentives may invoke quality reduction and under-servicing, in the NZPHCS where no such constraints apply, service providers will likely respond simply by passing on their increased share of financial risks via increased costs to patients. Such actions effectively 'undo' the benefits of patient risk pooling that in the first place provide the rationale for patients to join an insurance-based managed care scheme. All of the additional costs of an insurance scheme are incurred, but few of the cost-constraining tools of managed care are available. Indeed, such a scheme is likely more costly than a universal indemnity-based insurance scheme. Practitioners can recreate fee-for-service remuneration, so are left no worse off, but patients are worse off as they must endure restrictions in practitioner choice, service quality and service quantity, as well as paying the higher costs of treatment and institutional administration and monitoring than they would under the counterfactual.

Given the contractual limitations posed by the ability for general practitioners to charge patients directly, and strong general practitioner representation in the governance of PHOs, it is not surprising to find that most of New Zealand's seventy-seven PHOs as at December 2004 are undertaking no active insurance-based fiscal risk management activities. Financial risk management is not mentioned at all in the Ministry of Health-commissioned 2004 review [50] of PHO management services. An examination of the financial accounts of five PHOs affiliated to a general practitioner-owned management company in Wellington confirms that all capitation monies are being paid directly to either the practitioners (the component identified in Table 1 as GMS/Nurse Subsidies) or the management company (the payments identified in Table 1 as Services to Improve Access (SIA) Subsidies and per-capita management fees), with the PHOs undertaking no financial risk management activities [51]. Management fees and SIA payments are being used to fund the practice-based managed care strategies that PHOs are undertaking. PHOs are thus 'passing on' all the financial risks consequences of variations in patient demands under the NZPHCS arrangements are vested in the PHO-insurers to individual service providers by 'passing on' the capitation funding in its entirety.

### Service Providers Become Insurance Companies

PHO 'passing on' contracts are in effect turning the service deliverers into the 'insurance hubs' of the NZPHCS. Even though it is the service providers who now receive the government-funded GMS/Nurse subsidies, the payments are still insurance premiums and must be managed as such. The subsidies cannot be considered in any way equivalent to the 'Section 88' payments. In any given period, service providers must now manage the demands of all insured individuals – that is, their entire registered patient base – and the costs that a subset of them will incur. This means that service providers should be undertaking the task of determining the actuarially-fair premiums to charge each registered individual and recouping the costs from them all. However, service providers are not trained or qualified in the operation of insurance companies, and they do not typically have mechanisms in place for collecting remuneration unrelated to service consumption. A service providers' interaction with registered individuals is typically confined to the episodic interaction that occurs when an individual consumes care. Given the pre-NZPHCS practice of charging co-payments only to those individuals who consume care, it is not surprising to find that, once again, for administrative convenience, the pre-NZPHCS business model of patient charging only upon consumption of services is being used to recoup premium top-ups in the NZPHCS.

#### Risk-Rated Insurance Premiums

Utilising pre-NZPHCS arrangements to collect premium top-ups invokes some significant distributional consequences. Whereas pre-NZPHCS, the demand variations and associated risk management costs for the small subset of the population who received subsidies were shared jointly by all taxpayers, and self-insuring individuals faced the costs of variation only in respect of their own individual demand, under the NZPHCS, as payments are collected in the form of patient charges at each consultation, the variations associated with the demands of all 3.7 million registered New Zealanders and the risk management costs associated with the very much larger insurance scheme are borne only by that subset of the registered population that consumes health care.

Furthermore, as the levy is collected upon each treatment consumed, the more treatments an individual consumes, the more of the additional costs intended to be borne by the provider and the PHO (and by extension the wider insured population), are paid by the individual patient seeking treatment. Rather than sharing risk management costs amongst the entire population as intended by an insurance scheme, the charging instruments of the NZPHCS, and specifically the 'grandfathered' 1938 charging agreement, ensure that these costs are paid only by the sick. Indeed, the result is that the frequently sick pay a perfectly risk-rated individual insurance premium contribution that is determined by their actual health state, as it is perfectly correlated with their instances of demand for care. Risk-rated insurance premiums vary the premium paid by an individual based upon the amount of risk an individual brings to the scheme. Sicker individuals bring more costs as they consume more care. In a community-rated system, all individuals pay the same premium, irrespective of their individual likelihood of consuming (based upon the 'community' or 'population-based' average likelihood of consuming). When an individual who is riskier pays a higher premium than a less risky individual, this is termed risk-rating, as the individual likely to cause more cost pays more of the costs of the pool. As a sicker individual consumes more treatments, more co-payments will be made. Premium top-ups are higher for sicker individuals, therefore the premium top-up under the NZPHCS is risk-rated.

Meanwhile, the infrequently sick enjoy lower charges for health care due to increased subsidies under the NZPHCS, so are more likely consume more care than previously, in the form of 'over-consumption by the worried well'. These increased costs are added to the charges levied by providers on consuming individuals, leading to even higher charges to the sick, which are borne disproportionately by the more frequently sick, who are less likely to inefficiently over-consume in response to increased subsidies as they are more likely to be genuinely ill.

#### Risk Redistribution and Equity

Whilst it is recognised that under the pre-NZPHCS, some individuals paid the full cost of their primary health care, and with constrained resources there will be a requirement for some patients to continue to make some contribution towards their health care costs, the equity of recouping the highest charges from the lowest-subsidised, sicker-than-average individuals, with the burden on these individuals rising substantially as more individuals of other subsidy classes receive higher subsidies over time, irrespective of any change to those individuals' health states, warrants consideration.

Pre-NZPHCS, the self-insuring majority faced no costs related to the consumption behaviour of services by other individuals except via taxation. Under the NZPHCS, they bear the financial consequences of changes to both the consumption behaviour of all other patients registered at their practitioner, and the financial consequences of the lack of behaviour-altering incentives resulting from capitation 'passing on' and patient charging, directly in their payments when consuming. Due to their financial status (i.e. aged 24–64 years and not living in areas identified as 9 and 10 in the New Zealand Deprivation Index), previously self-insuring individuals are also most likely to be paying the higher taxation required to fund higher premium subsidies. The burden of the additional risk management costs will be highest on the last group of previously self-insuring individuals to receive higher subsidies, and will be especially acute if the subsidy increases are accompanied by ill-informed regulatory restraint on the payments made by the newly-subsidised in order to keep constant the sum of their subsidy and their co-payment, without consideration of the additional costs associated with the subsidy increase. Under these circumstances, over time, previously self-insuring individuals can expect to pay substantially more to a subsidised provider than to a provider who eschews the subsidised system entirely.

To date there is very little evidence of providers eschewing the subsidised scheme. Therefore, there will be very little information available to either regulators or patients to determine what constitutes a 'fair' charge for services independent of the charges associated with financial risk-bearing. If provider collectives can utilise their professional membership status to limit the extent of competition from unsubsidised practitioners, then competition from unsubsidised practitioners is likely to be weak, and the likelihood of ill-informed regulatory restraint substantial. [Anti-competitive behaviour by medical practitioners has previously been found in respect of the members of the Ophthalmological Society of New Zealand found guilty under Section 27 of the Commerce Act 1986 for refusing to register foreign ophthalmologists to practice in New Zealand in order to carry out cataract surgery at lower remuneration from government contracts than the current incumbents collectively agreed to provide the surgery [52]. Moreover, in the absence of an unsubsidised sector with the ability to signal the costs of service provision independent from risk management, the government will have little information upon which to base the setting of its own subsidy contributions. This situation is very different from England, where the vibrant private sector stands as a benchmark against which government can assess both the size of subsidies offered to, and the quality and quantity of services provided by, NHS entities.

### High Prices in Evidence

Evidence of higher patient co-payments under the NZPHCS, relative to its predecessor regime, is provided by the Consumers' Institute. Higher patient charges are reported in Interim PHOs than in Access PHOs, even for those individuals who generate identical subsidies under the two funding regimes [53]. This finding is consistent with the greater burden of additional risk management costs falling on practices with a larger number of lower-subsidised registered individuals. Rather than impose all the additional costs on the lower-subsidised individuals, these practices are opting to spread the higher costs amongst all patients. The Independent Practitioners Association chief executive has acknowledged that higher charges to patients are due to the increased financial risk that he claims practitioners are bearing. However, that patient charges have increased indicates that the practitioners are not absorbing the higher financial risks within their businesses, but are passing them onto patients.

That the burden of additional costs under the NZPHCS is real, significant and greatest upon frequently-ill low-subsidised individuals is in part reflected by the introduction, on 1 July 2004, of Care Plus. This additional capitated subsidy is designated to meet the additional costs incurred (initially to their practitioners, but ultimately passed on in higher patient charges) by frequently ill individuals who are not sufficiently sick to qualify for the higher subsidies available to high-use patients consuming twelve or more treatments in a twelve month period: "It's aimed at people who need to visit their family GP or nurse often because of significant chronic illnesses such as diabetes or heart disease, have acute medical or mental health needs, or a terminal illness" [54]. If the original capitation subsidies and population-based funding formulae had fairly allocated the costs of the system amongst individuals based upon patient need, and PHOs and practitioners had entered into contracts that minimised financial risk bearing costs and allocated the additional costs equitably across all patient classes, then arguably a Care Plus-type adjustment less than two years into the operation of the NZPHCS should not have been necessary. That the first substantive adjustment to the NZPHCS contracts addresses the costs of the category of patients that the foregoing analysis predicts will be most disadvantaged provides some strong circumstantial evidence supporting the analysis of this paper.

### Patient Pool Size Cost Implications

The full extent of the inequitable allocation of additional costs under the NZPHCS model is not restricted solely to the costs of increased consumption associated with increased subsidies and the absence of effective cost-sharing to induce providers to constrain their cost-causing behaviour. The inability to effectively incentivise service providers that results in PHOs 'passing on' capitation contracts to service providers, results in the service providers becoming the effective insurance providers. Their insurance pools therefore have very small numbers, so will almost certainly lead to greater variation of profitability between providers than would occur with larger patient pools. Furthermore, patients with identical health states in different patient pools will incur substantially different premium payments, simply because of the wide distribution of patient health states between insurers with very small pools. Thus, the NZPHCS will be even more costly and even less equitable than both a standard managed care scheme and the pre-NZPHCS arrangements.

#### Large Numbers, Insurer Profitability and Pool Management

Insurance systems rely upon the 'law of large numbers' to reduce the variations in insurer profitability resulting from the unequal distribution throughout the population of the characteristics that cause the insurer to incur costs. In the case of primary health care, the cost-causing event is a patient developing a condition that causes the patient to seek primary health care treatment. Assuming the likelihood of an individual requiring treatment in any one period is random, any given pool of patients will have a pool 'average likelihood of requiring treatment' that is either higher or lower than the population average. The smaller the pool, the greater is the likelihood that the pool average will be substantially different from the population average. However, the larger the pool, the greater the likelihood that the pool average will be close to the population average. When insurer revenue is determined by population averages, but the costs are determined by pool averages, in any given period half the pools will have costs in excess of revenues (incur losses) and half will have costs less than revenues (incur profits). The smaller the pool, the greater the probability of making a profit or loss substantially different to that of a pool with the population average.

Typically, if demands between periods are random and unrelated, then over time each pool will incur a random number of profits and losses that cancel each other out. Where a pool makes a loss, it is covered either by profits retained from the past, or some other financing (e.g. owner underwriting or reinsurance). Managing cashflows between periods thus incur costs – known as risk management costs. Managers of insurance pools seek to maximise profits, which leads to incentives to minimise risk management costs. For random pools, merging pools to obtain a profit closer to the population average will reduce the costs associated with managing cash flows in the event of a loss. The optimum pool size may therefore be quite large. However, if the demands are correlated, either that in one period one pool has a known greater likelihood of incurring a profit or a loss, or that demands of a pool across time are linked to demands in past periods, then the allocation of costs will be correlated and the pool will be either habitually profitable or habitually loss-making. If an insurer knows the pool is habitually profitable, then profits can be routinely extracted. Such an insurer has no incentive to merge the pool with a pool of unknown costs, as profitability may be reduced. However, if the pool is habitually loss-making, then the insurer faces an incentive to merge with other pools in order to reduce the probability of making a loss. Merging pools in this manner leads to larger numbers and a reduction in the likelihood that any one pool is substantially different from the population average. However, if there is any possibility that an insurer with a known low-cost pool can resist merging with other pools, the 'average' profitability of the remaining pools will be less than the population average including the highly profitable pool [55].

The incidence of health costs is likely to be correlated, both within and between time periods. Specific individuals with similarly low costs and similarly high costs may patronise similar insurers (e.g. individuals within a community may all get sick simultaneously, or an entire family with genetically linked high health needs may seek cover from the same company), and there is substantial evidence that a very large proportion of health costs are incurred by a very small number of individuals, even in respect of primary care health care demands [56,57]. This suggests that the variation in health pools is significant. United States data suggests that even where there is information on an individual's past consumption of health care, only "an estimated 20% to 25% of total variation in health care expenditures on an individual basis is predictable, and the remainder is random" [58]. This suggests that the optimal size of a health insurance pool will be large in order to manage the risks of the insurer incurring losses, and to reduce the costs of risk management. The United States Health Care Financing Administration considers capitated primary health care physicians or physician groups to be at substantial financial risk if they have fewer than 25,000 patients, whilst "primary care physicians may find capitation disadvantageous even if they have only one or two patients who happen to require intensive medical care during a given year, or have a consistently sicker panel of patients relative to other primary care physicians" [59].

#### Pool Size of New Zealand Insurers

In New Zealand, private sector general practitioners are typically sole practitioners. Even though they may share some common overheads via a 'group practice', including clinic space and reception services, each practitioner usually maintains an individual, independent practice based upon a 'patient list' that contains typically between 1200 patients in a rural practice and 2000 patients in an urban practice. Whilst PHOs notionally provide insurance cover for patient bases of between 3000 and 300,000 individuals [60], the 'passing on' of capitation payments results in effective insurance pools being managed at the level of each individual general practitioner – that is, insurance pools of between 1200 and 2000 individuals. Given the United States evidence, these numbers are likely to be far too small to efficiently manage the demand variation that will occur even with a random distribution of health states amongst the population. Profits will be larger and losses larger than if pools were larger. The costs of risk management will therefore be greater than if the pools were larger.

Loss-making practitioner-insurers in New Zealand face few incentives to merge their pools to reduce risk management costs as would occur in a typical insurance market, as their first resort is to recoup losses simply by charging patients for the difference between costs and PHO subsidies. The system offers no other incentives to manage the pool efficiently, so the greater profit variations, and their associated costs to patients, will persist largely unchecked. As the pools are small and profitability variations great, there will therefore be substantial variations in patient co-payment prices amongst providers, simply because of the variations in the underlying patient health states of the patient list. That is, if the insurer-provider has a 'high cost' pool, patient co-payments will be higher, even for a low-demand individual, than those of a provider with a population average pool, simply to recover the additional costs of variation in the demand of the pool from the capitated population average. The patient's payments are therefore determined by the risk level of the provider's pool – a patient of a given health state will pay different prices at different practices simply because of the 'luck' that determines the practitioner's risk profile relative to the population average. In the presence of very small pools, variations between individual provider pools even within a single subsidy level may be substantially greater than the population-based variations upon which the differential capitation premium subsidies (Interim and Access) of the PHO are based, leaving individual providers subject to returning very large losses or very large profits. Profitable insurer-providers will be able to extract profits in excess of costs as dividends, and even charge co-payments similar to those of competing high-cost providers, not because of any effort on the insurer-provider's part, but simply because of the 'luck' in allocation of patients and health care demands.

#### Pool Management and Provider-Insurer Competition

Normally, a patient facing a high patient charge will seek to shift custom to a lower-charging provider. However, a profitable, 'low-cost' NZPHCS provider will face few incentives to register a patient who responds to the prices of the 'high-cost' pool by seeking to transfer to the 'low-cost' pool, simply because that patient's past patronage of a 'high-cost' pool suggests that the patient is more likely to have higher-than-average demand characteristics. A 'low-cost' provider would prefer to engage in screening behaviour to assess the health state of the patient to ascertain that the patient's health state is at or better than the provider's current average before making an offer to the patient to join ('cream-skimming'). High-cost pools, on the other hand, face opposite incentives. Any transferring patient may actually improve the pool average, so is less likely to be 'screened out'. Hence, where such pool management occurs, the system equilibrium tends towards a small number of very large high-cost pools and many small low-cost pools, as occurs in the United States managed care market with large state-funded Medicare and Medicaid pools, and many smaller privately-funded managed care entities. In the New Zealand environment, over time this could manifest as a large number of small private, for-profit pools, and a few very large pools more likely to be operated by private, nonprofit providers who are less motivated by the profitability of the pool than by the nonprofit's objective to serve individuals. The larger pools, however, will be the more costly ones. If premium subsidies are adjusted over time based upon the costs of the higher-cost pools, then the smaller lower-cost pools will become even more profitable.

Furthermore, efficient operation of the insurance market requires provider-insurers to know, in respect of the risk profile of their pool, whether a surplus in any one year is a profit, which can be extracted, or simply a surplus required to be held to underwrite losses in future years. Specialist actuarial knowledge is required to make such judgements: "since providers are exposed to exogenous risk, efficient risk pooling requires reinsurance for providers" [61]. Operating general practices under the pre-NZPHCS assumptions that any surplus in a given year is a profit that can be extracted under the NZPHCS conditions where general practices are insurers will inevitably result in the extraction of funds that should have been applied to risk management, leading to the likelihood of even greater losses in the future, simply because the surpluses that would ordinarily have been retained to manage the risks of future losses have been extracted as dividends by for-profit providers. Thus, contrary to expectations, the NZPHCS will not "guard against funds being diverted from health gain and health services to shareholder dividends" [62]. Rather, the structures and relationships that have emerged have actually made it more likely that such activities will occur, even if inadvertently, because general practitioners who do not have the skills or experience to act as insurers have, as a result of the changes in funding and evolution of contracts under the NZPHCS become charged with the insurance task.

#### Provider Information and Pool Management

Moreover, if there is any additional potential for providers to utilise information about patients to 'select' their patients to manage costs, the risk management overhead of the NZPHCS will be even greater than under the counterfactual of a standard, separate-insurer managed care model. Arguably, given that only around 25% of cost variation is predictable, such selection may be difficult to achieve. However, of the small amount of variation that is predictable, past consumption of health services provides the best indicator [63]. This suggests that if active pool selection can be undertaken by insurers, access to an individual's past consumption information provides the best potential for selection to occur. Provider-insurers are arguably the best-placed to practice selection on this basis as they have access, via medical records, to existing patients' past medical history.

Reported actions of NZPHCS health service providers declining to take people 'on the books' (i.e. as an insured individual) but providing care as a 'casual' patient [64] could be interpreted as either an act of screening in order to avoid taking on the risks of a patient with unknown health state until an assessment has been made, or an act of deliberately declining a patient of probable high demand based upon past registration. In either case, patients are being denied coverage by the insurer of their choice, even though that insurer, in the capacity as a service provider, is happy to provide casual treatment. Given that the risk to the provider from a casual treatment is less, by the foregoing reasoning, casual treatments should cost less to deliver than subsidised treatments, so the cost savings could be passed on to patients. However, few providers will be likely to offer such differentiated prices, as low-subsidised patients willing to self-insure would likely face lower costs under such a system, so would respond by eschewing the subsidised system and opting for casual treatment, thereby reducing both the possibility of the low-cost practitioner extracting profits in excess of those feasible at the population average, and the ability for providers to recoup risk-management costs disproportionately from the highest co-paying class of patients.

## Conclusion

The preceding analysis indicates that the arrangements that have emerged under the NZPHCS, whilst in principle a managed care insurance system, are substantially less than optimal in respect of the requirements for a fully-functioning, efficient managed care insurance model. It appears that the use of institutions and relationships that prevailed under the pre-NZPHCS system in an 'evolutionary' move towards what appears to be a fully-fledged managed care model are likely to be counter-productive to the equity objectives of the strategy, substantially more costly than either the pre-NZPHCS system or the optimal managed care model, and as a consequence of the limited competition that exists, unlikely to respond to the normal competitive pressures to evolve into a more cost-efficient model. Thus, rather than the institutions and contracts being a 'first step' in an evolving strategy, the current higher-cost institutions and contracts are likely to become entrenched, to the long-term detriment of both taxpayers and patients.

Two instruments appear to be critical in the inability of the NZPHCS to achieve its full range of managed care objectives. The first is the presumption that general practitioners would retain their individual right to set patient co-payments independent of PHO contracts. This presumption has left PHOs with no meaningful ability to practice financial risk management. Whilst there is no policy statement about the retention of the right to charge, the unstated assumption that it exists has restricted the use of fiscal strategies associated with traditional managed care models. However, even if no such assumption existed, the second instrument, the requirement that service providers be part of PHO governance, means that providers have been granted sufficient power to ensure that the contracts under the NZPHCS do not leave them any worse off than pre-NZPHCS. In their capacity as PHO governors, general practitioners would be unlikely to be party to designing contracts that limit the professional autonomy that has been their non-negotiable bottom line in the development of New Zealand primary health care policy since 1938. As successive governments have been either unable or unwilling to restrain general practitioner charging autonomy using legislative powers, it is unlikely that PHOs, most of which are operating as general practitioner supplier-owned co-operatives, would be able to achieve such restraints using only mutually agreed contractual mechanisms.

Given that the outcomes of the NZPHCS are largely predictable from an analysis of risk-bearing in health insurance markets and the New Zealand history and institutions, the advisability of instituting a full, insurance-based managed care model with the associated requirements on the insurance companies to manage costs in a health care market that effectively denies to these managed care insurance organisations half of the tools normally available to such organisations to manage their costs must be questioned. The higher costs and inequitable distribution can be observed even at this early stage and will become more substantial over the next four years as premium subsidies increase. Indeed, the full extent of the higher costs at an individual level are likely being masked in the early stages by the sheer size of the additional government funding injected into the sector. As unrestricted practitioner charging negates the effects of patient demand pooling that normally accompany insurance schemes, any attempt to set up a managed care system based upon capitation payments of either the insurer or the service providers appears to be fundamentally flawed. This is not to say that practice-based strategies associated with PHO management and services to improve access cannot be legitimately and effectively funded on a capitation basis and provide measurable benefits. Indeed, benefits may have already accrued from these strategies. Rather it is a commentary on the wisdom of using capitation payments to fund the service delivery components of a universal insurance scheme in the presence of practitioner price-setting autonomy. Under current arrangements, the practice-based strategies would have to be extremely effective to outweigh the substantial additional costs of the imperfect insurance instruments that attend the NZPHCS in order to deliver a system that is of net greater benefit or offers better value-for-money than its predecessor.

In principle, leaving aside the power and professional autonomy of medical practitioners, the structures and intended contracts in the NZPHCS offered a potentially viable model of competing insurers and competing service providers that had the potential to deliver an efficient and effective insurance-based primary health care system for New Zealand that was capable of real innovation in both contracting and service delivery. However, to do so would have required a truly competitive environment, both in respect of insurance and service delivery markets, with fully transferable insurance premiums independent of insurer-service delivery contracts, and where insurers were governed, managed and operated as specialist insurance companies [65]. Such competition in the market for purchasers of services, where patients could freely exercise their insurance custom, would lead to genuine competition in the markets for both insurance customers and service provision contracts, where it would be harder for provider co-operatives to unilaterally determine the terms and conditions under which they would enter into contracts with insurers. This would have led to vibrant competition not just for contracts, but also in the models of insurance and care delivery that could move beyond the managed care model under which the system was established to alternative arrangements. If such a system allowed patient co-payments determined by the insurer, it would resemble the United States managed care model, but with the potential to evolve if models other than managed care proved more efficient. Such innovation is occurring in the United States as managed care proves less desirable for some patients and their insurers [66]. If patient co-payments were not allowed, then the New Zealand system it would resemble England's NHS. However, this model would require full funding from government sources.

In its present state however, the NZPHCS resembles neither of these models. It allows for all the additional costs of an insurance-based system, but none of the equity benefits of a fully state-funded system, and none of the fiscal benefits of a managed care system that constrains some of the excesses of insurance-based systems. Whilst there may be gains from the practice-based managed care strategies currently undertaken by PHOs, the costs of these gains will not necessarily be able to compensate for the substantial extra costs of the system as it is currently operating. If the costs and inequities of the NZPHCS escalate as predicted, any gains may be quickly eroded. Unless the deficiencies of the current insurance-based system are addressed soon, the very substantial proportion of the additional government funding committed to primary health care will likely amount to an ill-judged, overly-costly investment.

## Competing interests

The author(s) declares no competing interests.
